# Cold Pulsatile Machine Perfusion versus Static Cold Storage in Kidney Transplantation: A Single Centre Experience

**DOI:** 10.1155/2019/7435248

**Published:** 2019-01-16

**Authors:** Maria Irene Bellini, Sotiris Charalampidis, Paul Elliot Herbert, Vasileios Bonatsos, Jeremy Crane, Anand Muthusamy, Frank J. M. F. Dor, Vassilios Papalois

**Affiliations:** ^1^Imperial College Renal and Transplant Centre, Hammersmith Hospital, Imperial College Healthcare NHS Trust, London, UK; ^2^Department of Surgery, Imperial College London, London, UK

## Abstract

**Introduction:**

We present our experience with hypothermic machine perfusion (HMP) versus cold storage (CS) in relation to kidney transplant outcomes.

**Methods:**

Retrospective analysis of 33 consecutive HMP kidney transplant outcomes matched with those of 33 cold stored: delayed graft function (DGF), length of hospital stay (LOS), estimated glomerular filtration rate (eGFR), and patient and graft survival were compared. Renal Resistive Indexes (RIs) during HMP in relation to DGF were also analysed.

**Results:**

In the HMP group, mean HMP time was 5.7 ± 3.9 hours with a mean cold ischaemic time (CIT) of 15 ± 5.6 versus 15.1 ± 5.3 hours in the CS group. DGF was lower in the HMP group (p=0.041), and donation after Circulatory Death (DCD) was a predictor for DGF (p<0.01). HMP decreased DGF in DCD grafts (p=0.036). Patient and graft survival were similar, but eGFR at 365 days was higher in the HMP cohort (p<0.001). RIs decreased during HMP (p<0.01); 2-hours RI ≥ 0.45 mmHg/mL/min predicted DGF in DCD kidneys (75% sensitivity, 80% specificity; area under the curve 0.78); 2-hours RI ≥ 0.2 mmHg/ml/min predicted DGF in DBD grafts (sensitivity 100%, specificity 91%; area under the curve 0.87).

**Conclusion:**

HMP decreased DGF compared to CS, offering viability assessment pretransplant and improving one-year renal function of the grafts.

## 1. Introduction

Worldwide, the increasing demand for renal allografts and growing waiting lists has led to the utilization of organs through donation after Circulatory Death (DCD), although these organs are associated with higher rates of discard, retrieval associated injury [1], and up to 50% delayed graft function (DGF) in comparison to transplanted organs from donors after Brainsteam Death (DBD) [2].

In order to achieve the optimum outcome from each donated kidney and increase the survival benefit compared to the dialysis population [3], optimal organ preservation remains one of the major challenges to reduce current DGF rates [4] and the relevant detrimental long-term impact [5]. DGF is a well-established risk factor associated with reduced long-term graft and patient survival [6]; furthermore, recipients transplanted with kidney grafts that develop DGF face prolonged hospitalization and the overall relevant increased costs [7]. Promisingly, the use of machine perfusion technology has been associated with improved DGF rates, particularly for DCD organs [4].

The process underlying DGF include several pathophysiologic mechanisms derived from the donor ischemic injury and inflammatory signalling; it is the clinical manifestation of the acute kidney injury which affects the transplant parenchyma and, subsequently, renal function [8]. Animal studies have demonstrated that one of the main benefits of using cold pulsatile perfusion for preservation, is attributed to the improved endothelial release of nitric oxide and reduced secretion of endothelin-1 [9], resulting in a renoprotective effect [10], not achievable with standard static cold storage. This effect in the renal microvasculature provides a unique platform for active organ reconditioning during HMP, expressed in significantly lower number of pathological lesions on kidney biopsies [10].

One of the flow parameters in HMP, the Renal Resistance Index (RI), has been previously identified as a marker of the whole-organ microcirculatory damage after the retrieval ischaemic injury [11]. Monitoring RI could also provide a real time evaluation of the organ recovery during HMP [12].

The aim of the present study was to evaluate the effect of HMP during kidney transplant preservation in comparison to static cold storage based on a single centre experience.

## 2. Patients and Methods

The study is a single centre retrospective cohort analysis of hypothermic machine perfused kidneys (RM3® Waters Medical System, US) transplanted from March 2012 to April 2018 versus cold storage only. It was conducted in accordance with institutional ethics regulations; since it was a retrospective chart analysis, no informed consent was required.

The case controls were matched on 1:1 basis according to graft type (DBD or DCD), donor age, cold ischaemic time (CIT), and number of Human Leukocyte Antigen (HLA) mismatches between donor and recipients.

As soon as the kidneys were delivered into our centre and there was an impediment to proceed immediately with the transplant, HMP was chosen as the preservation method.

The University of Wisconsin solution was used for HMP, at a temperature between 4-5°C and at an initial peak systolic pressure of 45 mmHg. After 30 minutes of cold perfusion, the pressure was held constant ≥ 40 mmHg. RIs were recorded to monitor kidney parenchymal recovery.

CIT was defined the time from the start of cold perfusion during organ retrieval to the time of reperfusion during the transplant, including the HMP time. DGF was defined as the need for dialysis within 1 week of transplantation with a perfused graft. Furthermore, we compared the mean Modification of Diet in Renal Disease estimated glomerular filtration rate (eGFR) [13] until day 365 from transplantation and the length of hospital stay (LOS) between the two groups. Graft failure, censored for death, was defined as permanent return to dialysis.

All the patients received a steroid sparing immunosuppressive regimen (7-day course of steroids) with alemtuzumab induction and long-term Tacrolimus (TAC) monotherapy (trough level, 5-8 ng/mL) and neither the renal replacement therapy nor the immunosuppression protocol of our centre changed over the last 10 years.

## 3. Statistical Analysis

Continuous variables are presented as mean ± standard deviation and were compared using one way ANOVA. Independent t-test was used to analyse RI trend during machine perfusion. The confidence interval was set to 95%, and* p* was considered significant at less than 0.05. We used a linear regression model with stepwise procedure to test which parameters were acting as independent predictors for DGF. A generalised linear model of univariate repeated ANOVA with post hoc Bonferroni correction was used to determine whether mean eGFR differed statistically significantly during follow up. A receiver operator characteristic (ROC) curve was constructed to investigate the predictive accuracy of RI for DGF.

Analysis was performed using SPSS (IBM SPSS Statistics for Windows, Version 20.0; IBM Corp, Armonk, NY).

## 4. Results

Sixty-six transplanted kidney outcomes were analysed. Donor and recipient demographics are shown in Table 1. No statistical difference was observed between the HMP and CS group baseline characteristics: mean recipient's age, cause of kidney failure, numbers of grafts from DCD and DBD donors, number of HLA mismatches between donor and recipient, donor's age and CIT.

The mean HMP time was 5.7 ± 3.9 hours; in the HMP group, the mean CIT of 15 ± 5.6 hours and 15.1 ± 5.3 hours in the CS group (*p*=ns).

In a linear regression model with stepwise procedure, DCD was an independent predictor for DGF (p<0.01) in the whole cohort, occurring in 24/66 kidneys (36%): 8/33 (24%) machine perfused and 16/33 (48%) cold stored (p=0.041). The DCD kidneys that developed DGF in the HMP group were 5/12 versus 10/12 in the CS control cohort (p=0.036), confirming a protective effect of the HMP preservation for the grafts retrieved from DCD donors.

The patients receiving grafts with subsequent DGF had higher length of hospital stay (LOS): 11.6 ± 5.8 days versus 29.1 ± 18.1 days (p<0.001).

**Figure 1:**
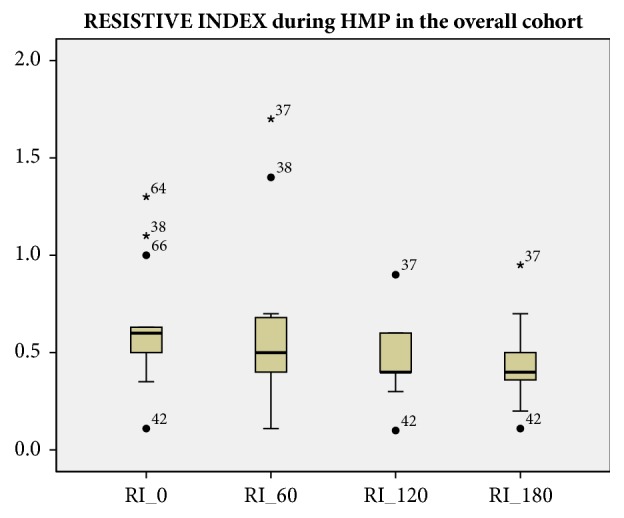
RI 0: Renal Resistive Index at the beginning of HMP. RI 60: Renal Resistive Index at 60 minutes of HMP. RI 120: Renal Resistive Index at 120 minutes of HMP. RI 180: Renal Resistive Index at 180 minutes of HMP. RIs are measured in mmHg/ml/min. HMP: Hypothermic machine perfusion.

**Figure 2:**
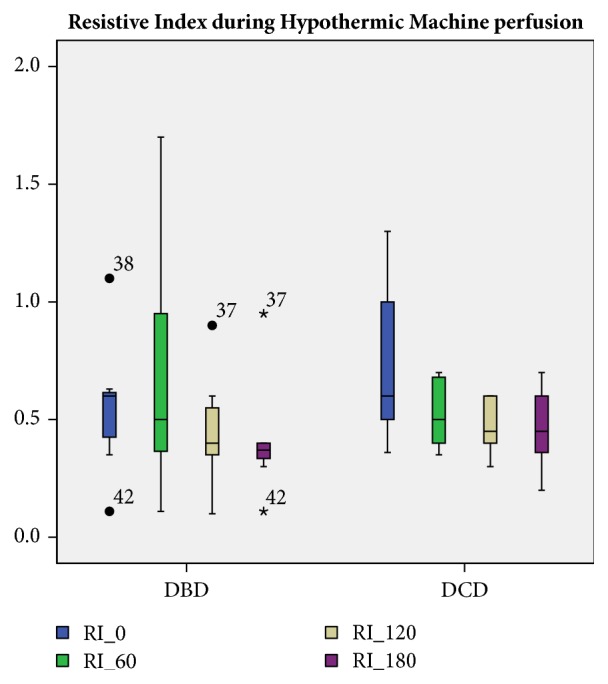
RI 0: Renal Resistive Index at the beginning of HMP. RI 60: Renal Resistive Index at 60 minutes of HMP. RI 120: Renal Resistive Index at 120 minutes of HMP. RI 180: Renal Resistive Index at 180 minutes of HMP. DBD: Donation after Brain Death. DCD: Donation after Circulatory Death. HMP: hypothermic machine perfusion. RIs are measured in mmHg/ml/min.

The RI decreased statistically significantly during HMP: mean RI at baseline (R0) was 0.65 ± 0.25 mmHg/ml/min (p<0.01); after 60 minutes (RI60) was 0.62 ± 0.33 mmHg/ml/min (p<0.01); after 120 minutes (RI 120) was 0.46 ± 0.16 mmHg/ml/min (p<0.01); after 180 minutes (RI180) was 0.44 ± 0.22 mmHg/ml/min (p<0.01). The higher impact in decreasing the original RI value was observed between the first and the second hour of HMP (p< 0.01). Figures 1 and 2 represent mean RI during HMP for the whole cohort, and the DBD and DCD subgroups.

**Figure 3:**
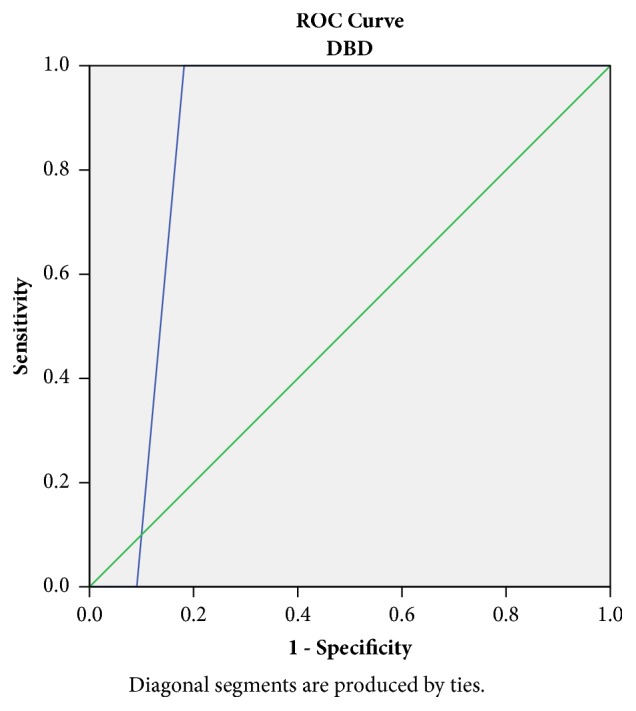
Receiving Operator Curve (ROC) for Resistive Index at 120 minutes (RI120) ≥ 0.2 mmHg/ml/min in DBD grafts: sensitivity 100%, specificity 91% in DGF prediction. Area under the curve 0.87.

Furthermore, we subanalysed the difference in RIs between DCD and DBD grafts. A 2-hours RI value ≥ 0.2 mmHg/ml/min was associated with 100% sensitivity and 91% specificity in DGF prediction for DBD grafts. The area under the curve was 0.87 (Figure 3).

**Figure 4:**
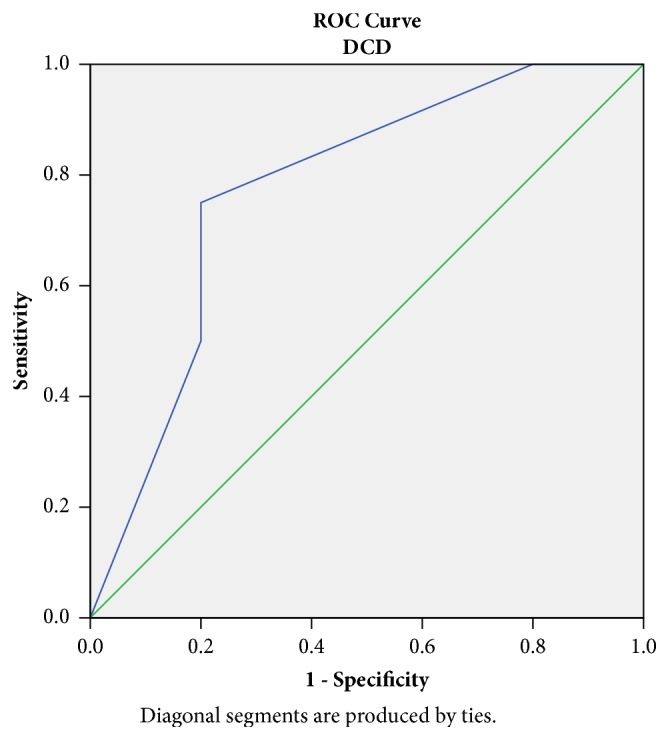
Receiving Operator Curve (ROC) for Resistive Index at 120 minutes (RI120) ≥ 0.45 mmHg/ml/min in DCD grafts: sensitivity 75%, specificity 80% in DGF prediction. Area under the curve 0.78.

A 2-hours RI value ≥ 0.45 mmHg/ml/min was associated with 75% sensitivity and 80% specificity in DGF prediction for DCD grafts. The area under the curve was 0.78 (Figure 4).

**Figure 5:**
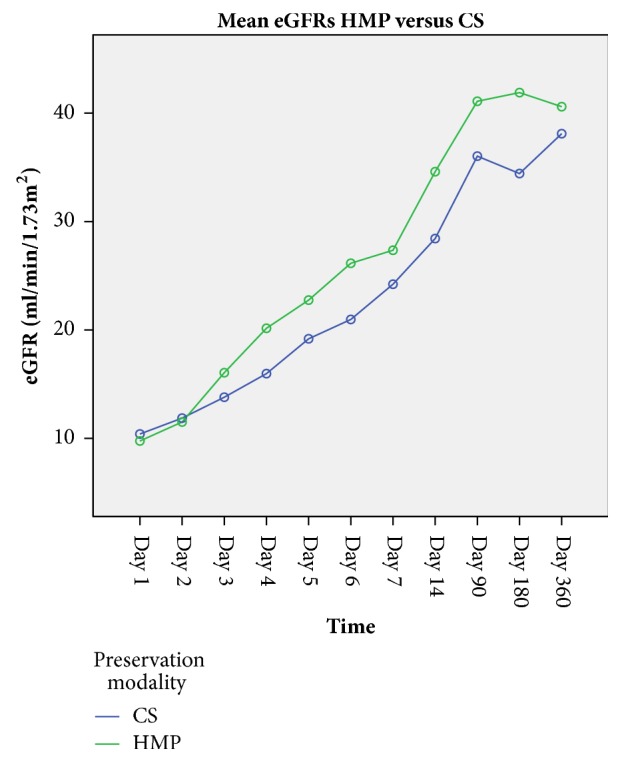
Generalised linear model of univariate repeated measures ANOVA. A total of 47 kidney recipients completed 365 days follow up and were analysed (20/33 HMP and 27/33 CS). Mean eGFRs were statistically different during follow up in HMP preserved when compared to CS kidneys (p= 0.039). Post hoc tests using the Bonferroni correction revealed that at day 365 mean eGFRs are higher in the HMP group (p<0.001).

Forty-seven patients had a transplant follow up longer than 365 days: 20/33 and 27/33 in the HMP and CS cohorts respectively. Multivariate analysis of univariate repeated eGFRs measures showed a statistically significant difference between the HMP and the CS groups (*p*=0.039), with the eGFRs for the HMP transplanted kidneys being persistently higher (Table 2). Post hoc tests using the Bonferroni correction revealed that higher values of eGFRs at day 365 were associated with HMP perfusion (p<0.001), Figure 5.

One graft loss occurred in the HMP group at 180 days due to acute rejection; 3 grafts were lost in the CS cohort: venous thrombosis (n=1) at 120 days posttransplant and acute rejection (n=2), at 180 days posttransplant (p=0.31). One patient died in the HMP group with a nonfunctioning graft after 270 days posttransplant due to myocardial infarction; no patient died in the CS (p=0.32). Results are summarised in Table 3.

## 5. Discussion

DGF is the Achille's heel of kidney transplantation from DCD donors, affecting more than half of the subsequently transplanted grafts [14]. Our findings demonstrated the protective role of HMP in this particular subcohort, related to reduced DGF rate. In our study, the incidence of DGF was higher in the CS compared to the HMP cohort (p=0.041). For transplants from DCD kidneys the incidence of DGF was lower in the HMP compared to the CS cohort (p=0.036).

The use of cold pulsatile technology is a long-established alternative to static cold storage and it has been shown to be a better preservation method [15]. There are different types currently available for clinical kidney preservation; our policy is to use cold pulsatile perfusion devices, like the RM3® because of its potential renoprotective effect [10]. This particular technology results in better preservation of the endothelial integrity and recovery, with improved endothelial release of nitric oxide and reduced secretion of endothelin-1 in ex-vivo models [9]. In this way, the underlying mechanisms of DGF are actively repaired, with a substantial difference from static cold preservation. In our study, the use of HMP resulted in the lower incidence of DGF, especially in the challenging DCD group, and higher eGFRs observed for HMP kidneys consistently during the 365 days of follow-up; this demonstrates the protective short- and long-term effect of HMP. Another advantage that the pulsatile technology provides is a platform during which the graft could be actively reconditioned, making it particularly attractive for higher-risk kidneys [16], as it delivers oxygenation, or any other nutrients or reconditioning agents, and creates a window of opportunity during which to assess the viability and quality of the graft before transplantation [17]. It has been previously shown that the RI is an independent predictor during HMP for the later development of DGF; however, it cannot be a stand-alone tool in predicting DGF, especially when considering the heterogeneity of the factors that can affect the transplant outcome [12].

Nevertheless, an important advantage of RI monitoring could be the ability to estimate the risk of a particular kidney to develop DGF [12]. RIs are known to rise in parallel to the development of parenchymal injury [18] and increased RIs are associated with donation after Circulatory Death and donor age [19]. In our study, the prevalence of the parenchymal damage in kidneys from DCD donors was demonstrated by the higher renal Resistive Index at 2 hours post HMP: 0.2 mmHg/ml/min for DBD versus 0.45 mmHg/ml/min for DCD. The relative ROC curves, associated with DGF incidence, had an accuracy of 87% and 78%, respectively (Figures 3-4). As previously reported, RIs are expression of the microcirculatory damage occurring within the parenchyma [11]; therefore the stress induced by the circulatory arrest is unsurprisingly linked to a worse profile.

Knowing the risk profile of a particular kidney earlier in the preservation process would be of great benefit for the postoperative management and it would provide objective information for selecting a particular recipient for a particular kidney, thus tailoring the offered renal replacement therapy to the patient who would benefit most. In the era of patient tailored consent [20] and patient centred outcomes, it is mandatory to involve the transplant recipient and allow him/her to consider the risks related to increased chances of DGF if transplanted with kidney for which there is evidence that it is in such risk. The present study showed that the HMP cohort had significantly higher eGFRs at 365 days of follow up when compared to the CS group. Thus, the impact of DGF in the long-term outcome is elicited by the difference in the preservation techniques, particularly for kidneys from high risk donors, like DCDs. We have also shown that DGF is associated with prolonged LOS, thus significantly impacting on patient morbidity and hospital cost; the advantages demonstrated in our study by the use of HMP are associated with better outcomes related to those important social and economic aspects [20].

## 6. Conclusions

Within the limitations of the size of the HMP and CS groups, our study demonstrated that hypothermic machine perfusion offers an advantage in deceased donor renal transplantation of high risk kidneys, since it reduces significantly DGF rates and is associated with higher posttransplant eGFRs. This preservation modality has a positive impact in kidney transplant outcomes from DCD donors and offers an early viability assessment that allows prediction of short- and long-term posttransplant graft function. It represents a real time opportunity to recondition the retrieval ischaemic injury, plan the postoperative recovery, and enhance the decision-making process by offering the patients evidence that allow them to make an informed decision.

## Figures and Tables

**Table 1:** Baseline recipients and donors characteristics. No significant difference between the two groups with ANOVA.

		Preservation Modality			
		Cold Storage		Hypothermic Machine Perfusion	
		Mean ± St. Dev	Total	Mean ± St. Dev.	Total

Recipient Age (years)		57 ± 10		55 ± 11	

Cause of kidney failure	Adult Polycystic Kidney Disease		3		1
	Diabetes Mellitus		9		11
	Glomerulosclerosis		3		4
	Hereditary Nephritis		1		1
	HIV Nephropathy		1		2
	Hypertension		3		4
	IgA Nephropathy		1		3
	Ischaemic Nephropathy		1		0
	Lithium toxicity		1		1
	Membranous Nephropathy		1		1
	Myeloma derived Nephritis		1		0
	Obstructive nephropathy after Rhabdomyolysis		1		0
	Systemic Lupus Erythematosus nephritis		1		0
	Tubulo-Interstitial Nephritis		1		0
	Unknown		5		5

Donor after Circulatory Death	N		21		21
	Y		12		12

HLA Mismatch	0		1		1
	1		1		2
	2		8		6
	3		12		12
	4		11		3
	5		0		2

Donor age (years)		58 ± 14		58 ± 14	

Cold Ischaemic Time (hours)		15.1 ± 5.3		15.0 ± 5.6	

HLA= human leukocyte antigen, N= no, and Y= yes.

**Table 2:** 

**Effect of Preservation modality in eGFR during follow up**					

Measure: eGFR ml/min/1.73m^2^					

Preservation Modality	Time	Mean eGFR	Std. Error	95% Confidence Interval	
				Lower Bound	Upper Bound

Cold Storage n = 27	Day 1	10.481	.978	8.511	12.452
	Day 2	12.000	1.350	9.281	14.719
	Day 3	14.000	2.274	9.421	18.579
	Day 4	16.296	3.257	9.737	22.856
	Day 5	19.593	3.687	12.168	27.018
	Day 6	21.407	3.982	13.386	29.428
	Day 7	24.667	4.100	16.409	32.925
	Day 14	28.000	3.555	20.840	35.160
	Day 90	34.370	3.379	27.564	41.176
	Day 180	32.926	3.347	26.185	39.667
	Day 365	36.630	3.443	29.695	43.565

Hypothermic Machine Perfusion n=20	Day 1	9.750	1.137	7.461	12.039
	Day 2	11.500	1.569	8.340	14.660
	Day 3	16.050	2.642	10.729	21.371
	Day 4	20.150	3.784	12.529	27.771
	Day 5	22.750	4.283	14.123	31.377
	Day 6	26.150	4.627	16.831	35.469
	Day 7	27.350	4.764	17.755	36.945
	Day 14	34.600	4.130	26.281	42.919
	Day 90	41.100	3.926	33.192	49.008
	Day 180	41.900	3.889	34.067	49.733
	Day 365	40.600	4.001	32.542	48.658

**Table 3:** Results. DGF was statistically significantly higher in the Cold Storage group and in DCD grafts. Length of hospital stay was longer in kidney that developed DGF (ANOVA).

				Preservation modality				*p value*
				Cold Storage		Hypothermic Machine Perfusion		
				Total	Mean ± St. Dev.	Total	Mean ± St. Dev.	

DGF	N			17/33		25/33		**0.041**
	Y			16/33		8/33		

DGF	N	LOS (days)		17/33	12.8 ± 6.4	25/33	9.9 ± 4.1	0.69
	Y	LOS (days)		16/33	36.4 ± 20.6	8/33	25.3 ± 16.1	
	N	LOS (days)		42/66	11.6 ± 5.7			**<0.01**
	Y	LOS (days)		24/66	29.1 ± 18.2			

DGF	N	DCD	N	15/21		18/21		0.27
			Y	2/21		7/21		
	Y	DCD	N	6/12		3/12		**0.036**
			Y	10/12		5/12		

Graft loss	N			30/33		32/33		0.31
	Y			3/33		1/33		

Pt survival	N			0/33		1/33		0.32
	Y			33/33		32/33		

DCD= donation after circulatory death; DGF=delayed graft function; LOS= length of hospital stay; N= no; Y=yes.

## Data Availability

The data used to support the findings of this study are included within the article.

## Abbreviations

CIT: Cold ischaemic time
CS: Cold storage
DBD: Donation after Brainsteam Death
DCD: Donation after Circulatory Death
DGF: Delayed graft function
eGFR: estimated glomerular filtration rate
HLA: Human Leukocyte Antigen
HMP: Hypothermic machine perfusion
LOS: Length of hospital stay
RI: Renal Resistive Index
RI 60: Renal Resistive Index at 60 minutes of hypothermic machine perfusion
RI 120: Renal Resistive Index at 120 minutes of hypothermic machine perfusion
RI 180: Renal Resistive Index at 180 minutes of hypothermic machine perfusion.
